# Milk fat globule membrane supplementation in formula modulates the gut microbiome and metabolic status of piglets and normalizes intestinal development

**DOI:** 10.3389/fnut.2025.1632519

**Published:** 2025-10-03

**Authors:** Zonghan Zhou, Bin Liu, Jun Shen, Caihong Yan, Guang Yang, Yu Zhang, Junying Zhao, Lijun Chen

**Affiliations:** ^1^Key Laboratory of Dairy Science, Ministry of Education, Food Science College, Northeast Agricultural University, Harbin, China; ^2^National Engineering Research Center of Dairy Health for Maternal and Child, Beijing Sanyuan Foods Co. Ltd., Beijing, China; ^3^Beijing Engineering Research Center of Dairy, Beijing Technical Innovation Center of Human Milk Research, Beijing Sanyuan Foods Co. Ltd., Beijing, China; ^4^Beijing Center for SPF Swine Breeding & Management, Beijing Commerce and Trade School, Beijing, China

**Keywords:** milk fat globule membrane, gut microbiota, metabolites, intestinal development, piglet

## Abstract

Milk fat globule membrane (MFGM) supplementation of infant formula demonstrates potential efficacy in modulating gut microbiota and metabolic profiles. However, the associated site-specific effects on intestinal microbial composition remain unclear. In this study, we used a neonatal piglet model to investigate the mechanisms associated with the metabolic regulation of supplemental MFGM and characterized the compartment-specific modulatory effects on intestinal microbial communities. A total of 20 piglets were randomly allotted to one of the following three groups: breastfed (BF), standard formula (SF), and MFGM-supplemented formula (EF). These diets were administered until weaning, with subsequent provision of commercial feed until euthanasia. Morphometric, microbial, and serum metabolomic analyses revealed that compared to piglets in the SF group, those in the EF group were characterized by significantly enhanced jejunal villus height (*p* < 0.05) and reduced cecal Oxalobacter (*p* < 0.05) and Pasteurella abundances, which were comparable to the levels detected in the BF group. Metabolically, piglets in the SF group demonstrated significantly lower levels of tyrosine, phenylalanine, and *β*-alanine (*p* < 0.05) and higher levels of 3-methyl-2-oxovalerate (*p* < 0.05) than those in BF piglets. In contrast, compared to the SF piglets, EF piglets exhibited significantly elevated levels of betaine (*p* < 0.05) and lysine. Spearman’s correlation analysis revealed significant positive associations between Oxalobacter abundance and creatinine, dimethyl sulfone, phenylalanine, tyrosine, and *β*-alanine concentrations, with inverse correlations observed for 3-methyl-2-oxovalerate and lysine levels. In conclusion, these findings revealed that MFGM supplementation contributes to maintaining a normal intestinal architecture, modulates site-specific microbiota, and mitigates metabolic disparities between formula-fed and breastfed neonates. Notably, these effects are primarily mediated via choline pathway regulation and competitive inhibition of pathogenic bacteria.

## 1 Introduction

The gut microbiome plays a crucial role in maintaining human health by regulating systemic physiological balance and is increasingly recognized as a key factor in the development of numerous diseases. Among these are conditions linked to metabolic and immune dysfunctions—such as obesity, asthma, allergic diseases, and diabetes mellitus—that have been strongly associated with microbial imbalance or dysbiosis in the intestinal environment (1–3). Importantly, early life, particularly the neonatal phase, represents a sensitive period for initial microbial colonization (4), Disruptions to this process, even if temporary, may predispose individuals to a variety of disease manifestations later in life (5–7).

Diet exerts a fundamental influence on the composition and development of the neonatal gut microbiome. Although the World Health Organization endorses breastfeeding as the optimal nutritional source for newborns, it is not universally feasible. As a result, enhancing infant formula to better replicate both the nutritional composition and biofunctional properties of human breast milk has become a pressing objective. Among the bioactive components receiving increasing attention is the milk fat globule membrane (MFGM), recognized for its beneficial effects on infant gut microbial ecosystems (8–10) and metabolic regulation (11–13). Structurally, MFGM is a complex trilaminar membrane encasing triacylglycerol-rich fat droplets in mammalian milk (14), consisting primarily of glycoproteins, phospholipids, sphingolipids, gangliosides, sialic acid, choline, and cholesterol (15–18). Notably, MFGM helps establish the intestinal microbiota in infants (8, 10, 19). Specifically, certain glycoproteins present in MFGM act as substrates for microbial fermentation, and the resulting metabolic byproducts influence the intestinal microbiota (15). Other MFGM constituents, such as phospholipids, gangliosides, lactadherins, mucins, and fatty acid-binding proteins, possess antimicrobial properties that inhibit pathogenic bacterial growth (18). In contrast, conventional infant formulas are typically derived from plant oils and lack these MFGM-associated bioactive molecules, which may partly explain the divergence in gut microbiota profiles between formula-fed and breastfed infants.

Differences in metabolic outcomes are also evident between these two feeding groups (13, 20, 21). For instance, variations have been documented in the concentrations of specific lipids (22), hormones (23), and amino acids. Moreover, infant formulas generally contain higher levels of protein than human milk. However, research indicates that even when protein content in formula is reduced by 20%, the metabolic phenotype of four-month-old infants remains largely unaffected (24). Therefore, MFGM could be pivotal for mitigating these disparities between BF- and formula-fed infants (11, 13, 15), although the precise mechanisms remain unclear.

Multiple animal models have been used to investigate the diverse beneficial effects of MFGM in neonates and the associated mechanisms (9, 25–27), among which, porcine models have been established to have greater physiological congruence with human infants than rodent models in recapitulating key features of postnatal intestinal development and nutritional physiology (28–30). Notably, MFGM positively affects intestinal barrier function in piglets (8). Despite these promising observations, the site-specific influence of MFGM on both the microbial landscape and metabolic activity within different intestinal regions in newborn piglets remains poorly characterized. This study, therefore, aims to explore the regional impacts of MFGM on gut microbial colonization and to elucidate its role in guiding neonatal metabolic development using a piglet model.

## 2 Materials and methods

### 2.1 Animal ethics statement

The Animal Welfare and Ethical Review Committee of China Agricultural University approved the experimental protocols (reference number CAU20160628-2), which were conducted following the approved guidelines. This study complied with the ARRIVE guidelines.

### 2.2 Animal and experimental design

Twenty specific pathogen-free (SPF) Yorkshire piglets (11 males and 9 females; average birth weight: 1.43 ± 0.29 kg) delivered vaginally from sows at the Beijing SPF Pig Breeding and Management Center were divided into three groups (6–7 piglets per group): BF, standard formula (SF), and experimental formula supplemented with MFGM (EF). Piglets in the BF group were housed with the sows and breastfed for 21 days after birth, after which they were fed commercial feed. Piglets in the SF and EF groups were breastfed for the first 5 days to acquire maternal antibodies, and from day five onwards, they were fed a standard formula (Sanyuan, China) or an MFGM-supplemented formula (19) (Sanyuan, China), respectively, until commercial feed was introduced at 21 days. The macronutrient compositions and function-specific components of the experimental formula are listed in Tables 1, 2, respectively. Throughout the experiment, all piglets had free access to water and were housed in a 12-h light–dark cycle (minimal lighting provided during the dark cycle) at 27 °C. On day 31, all piglets were anesthetized with 5 mL of ketamine (10 mg/mL) administered intramuscularly and then euthanized.

**Table 1:** Macronutrient compositions in standard formula (SF) and MFGM-supplemented formula (EF).

Items	Units	Content (per100g)
Energy	kilojoule (KJ)	2,162
Protein	gram	11.6
Fat	gram	27
Carbohydrates	gram	56.4
1,3-Dioleoyl-2-palmitoyl Triglyceride	gram	4.2
Sn-2 Palmitic Acid as Percentage of Total Palmitic Acid	%	40

**Table 2:** Function-specific components in standard formula (SF) and MFGM-supplemented formula (EF).

Nutrients	SF (g/100 g)	EF (g/100 g)
Total MFGM protein	Trace	0.251
Phospholipid	0.153	0.301
Sphingomyelin	Trace	0.0688
Sialic acid	0.085	0.171
Total lactoferrin	Trace	0.0344
IgG	Trace	0.172
Lactadherin	Trace	0.043
Ganglioside	Trace	0.0086
Mucin1	Trace	0.0602

SF, standard formula; EF, experimental formula with milk fat globule membrane supplementation.

### 2.3 Tissue collection

On day 31 of the study, the entire gastrointestinal tract was excised under sterile conditions. The contents from specific intestinal regions (including the duodenum, jejunum, ileum, cecum, and colon) were collected and immediately cryopreserved at −80 °C. Additionally, major organs such as the brain, heart, pancreas, and spleen were excised and weighed individually. To evaluate immune organ development, the spleen index was determined by calculating the ratio of spleen weight to total body mass (g per 100 g body weight). Segments of the small (duodenum, jejunum, ileum) and large intestines (cecum and colon) were fixed in 10% neutral buffered formalin, embedded in paraffin, sliced into 5 μm sections, and stained using standard hematoxylin and eosin protocols. Imaging was conducted using an Olympus BX50 light microscope (Olympus, Tokyo, Japan) equipped with a Leica DFC 320 digital imaging system (Leica Microsystems, Wetzlar, Germany).

### 2.4 Histological analysis of crypt and villus morphology

To assess intestinal morphology, ten well-oriented villi and their associated crypts were randomly selected per subject for measurement. Image analysis was performed using CellD software (Olympus), which enabled quantification of villus height (from the apex to the crypt junction), base width, and surface area, thereby providing histomorphometric insights.

### 2.5 Serum metabolites

#### 2.5.1 Sample preparation

Serum samples were centrifuged at 13,000 rpm for 15 min. From each sample, 450 μL of the aqueous supernatant was transferred into a fresh 2-mL microcentrifuge tube. To each aliquot, 50 μL of a 3-(trimethylsilyl)-1-propanesulfonic acid (DSS) solution (Anachro Technologies Inc., Canada) was added as an internal standard. The mixture was vortexed thoroughly and transferred to a 5-mm NMR tube (Norell, Morganton, NC, USA). Samples were kept at 4 °C and analyzed within 24 h.

#### 2.5.2 NMR data collection

Nuclear magnetic resonance (NMR) spectra were acquired using a DD2 600 MHz spectrometer (Agilent Technologies, Santa Clara, CA, USA) fitted with a triple-resonance cryogenic probe. A 1H-NMR spectrum was recorded utilizing the first increment of a two-dimensional 1H–1H NOESY pulse program to suppress water resonance. Acquisition parameters included a 100 ms mixing time and a 990 ms pre-saturation period (~80 Hz gammaB1). Spectra were obtained at 25 °C with 64 scans per sample, yielding a total acquisition time of approximately 7 min.

#### 2.5.3 Spectral processing and metabolite identification

Raw free induction decay (FID) signals were automatically processed using Chenomx NMR Suite 8.1 (Chenomx Inc., Edmonton, Canada), incorporating zero filling and Fourier transformation. Manual phase and baseline corrections were subsequently applied by a trained technician. Spectra were referenced to the DSS internal standard and matched against the Chenomx metabolite library. Across all analyzed spectra (*n* = 250), a total of 67 metabolites were identified and quantified in micromolar (μM) units. Data were normalized to the weight of each sample prior to statistical analysis, and any datasets suspected of contamination were excluded.

### 2.6 Microbial DNA extraction and 16S rRNA amplicon sequencing

Microbial genomic DNA was extracted from homogenized fecal matter using the QIAamp Fast DNA Stool Mini Kit (Qiagen, Hilden, Germany), following the manufacturer’s guidelines. The hypervariable V3–V4 regions of the 16S rRNA gene were PCR-amplified using primers 341F (CCTACGGGGNGGCWGCAG) and 805R (GACTACHVGGGTATCTAATCC). Each PCR reaction contained 10 ng of DNA, 15 μL of Phusion High-Fidelity PCR Master Mix (New England Biolabs, Ipswich, MA, USA), and 0.2 μM of each primer in a final volume of 30 μL. Thermocycling conditions consisted of an initial 3-min denaturation at 95 °C, followed by 25 cycles of denaturation (95 °C, 30 s), annealing (55 °C, 30 s), and extension (72 °C, 30 s). Amplicons were processed into sequencing libraries using the NEBNext Ultra DNA Library Prep Kit for Illumina (New England Biolabs), and paired-end sequencing (2 × 250 bp) was performed on an Illumina HiSeq 2500 platform (Illumina Inc., San Diego, CA, USA).

Raw reads were subjected to quality control, clustering, and chimera filtering using USEARCH v1.9 (23) and VSEARCH v2.6 (24). Sequences sharing ≥97% similarity were clustered into operational taxonomic units (OTUs), and taxonomic classifications from phylum to species were assigned using the RDP 16S rRNA training set (Version 16) (31).

### 2.7 Data analysis and statistics

Quantitative results are presented as means ± standard deviations, and visualizations were generated using GraphPad Prism 9.0 (GraphPad Software Inc., La Jolla, CA, USA). Group differences were assessed using one-way ANOVA or the Kruskal–Wallis test, with *post hoc* comparisons conducted via Dunn’s test. Adjustments for multiple comparisons were made using either Bonferroni correction or false discovery rate (FDR). Statistical significance was defined as *p* < 0.05.

Microbiome data were processed and interpreted using MicrobiomeAnalyst 2.0 (32). Prior to analysis, OTU abundances were normalized using total sum scaling. Alpha diversity metrics and intergroup comparisons were analyzed via the Kruskal–Wallis test followed by Dunn’s multiple comparisons. Beta diversity patterns were evaluated using principal coordinates analysis (PCoA) based on Bray–Curtis dissimilarity, and group separation was tested using permutational multivariate ANOVA (PERMANOVA). Taxa showing differential abundance at the genus level were identified using linear discriminant analysis effect size (LEfSe). For metabolomic profiling, data were log10-transformed and Pareto scaled, and statistical interpretation was conducted through the MetaboAnalyst 6.0 online tool (33).

## 3 Results

### 3.1 Effects of dietary type on body weight and wet tissue weight

At 31 days of age, the brains, hearts, pancreases, and spleens of the piglets were individually weighed, and corresponding organ indices were computed relative to body weight. Analysis revealed a significant influence of dietary intervention on spleen wet weight (see Table 3). Notably, piglets in the BF group exhibited a significantly greater spleen weight compared to those in the SF and EF groups (one-way ANOVA, *p* < 0.05). However, no statistically significant differences were observed in the wet weights of the brain, heart, or pancreas across the dietary groups.

**Table 3:** Effects of dietary type on the tissue wet weights of 31-day-old piglets (*n* = 4–5).

Item	Group		
	BF	SF	EF
Born weight, kg	1.57 ± 0.38^a^	1.55 ± 0.22^a^	1.47 ± 0.30^a^
D30 weight, kg	6.77 ± 1.12^a^	3.88 ± 0.68^b^	3.48 ± 0.70^b^
Brain, g	43.22 ± 3.09^a^	48.18 ± 2.31^a^	44.25 ± 3.56^a^
Heart, g	36.62 ± 5.18^a^	28.10 ± 2.82^a^	22.85 ± 7.01^a^
Pancreas, g	11.94 ± 4.74^a^	8.85 ± 1.82^a^	7.85 ± 2.22^a^
Spleen, g	13.48 ± 2.28^a^	7.10 ± 2.66^b^	6.25 ± 1.67^b^
Spleen index	201.78 ± 34.07^a^	178.84 ± 33.57^a^	177.78 ± 12.73^a^

^a,b^*p* < 0.05. BF, breastfed; SF, standard formula; EF, experimental formula with milk fat globule membrane supplementation.

### 3.2 Effects of dietary type on intestinal morphology

**Figure 1:**
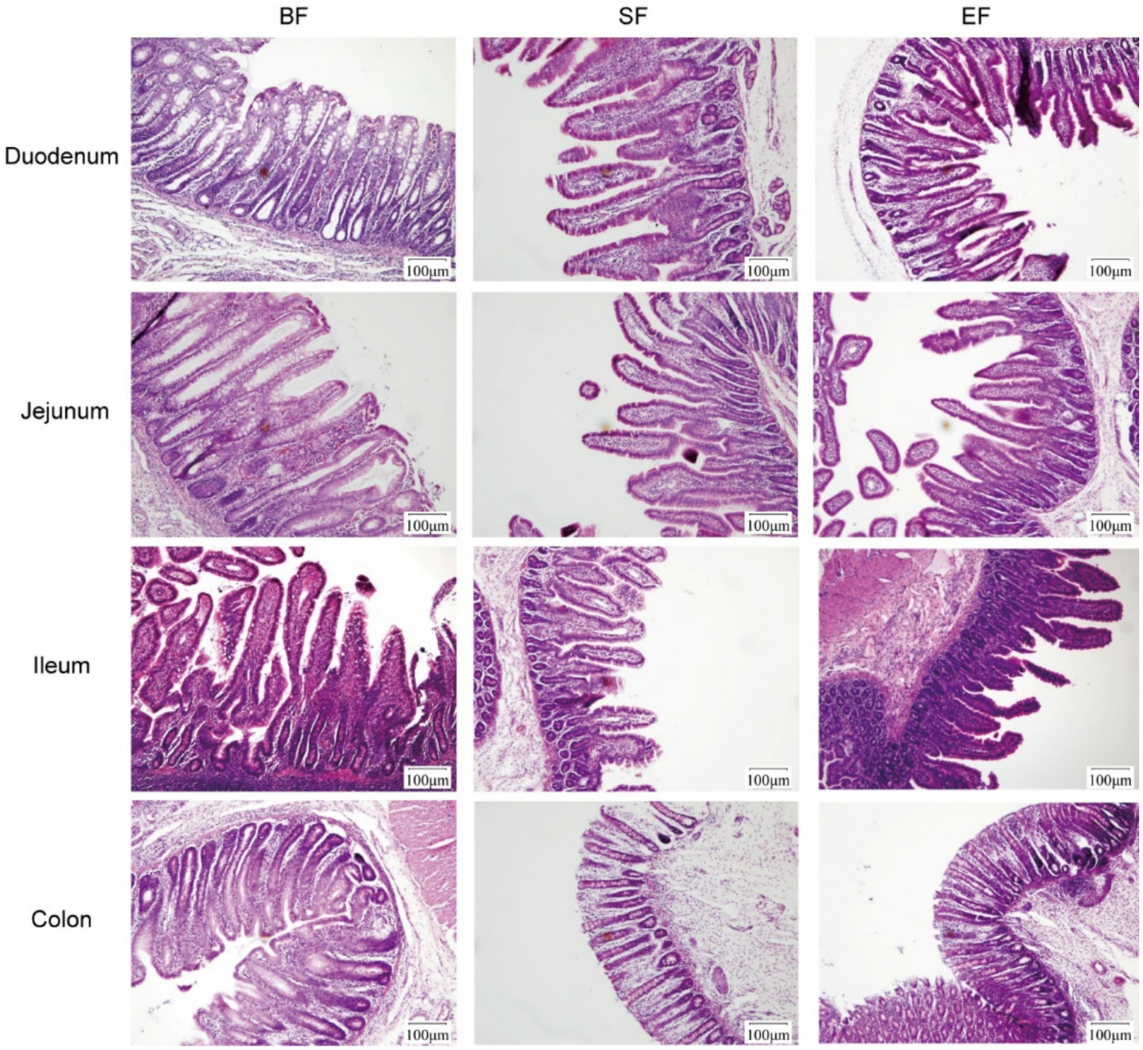
Effects of dietary type on the intestinal morphology of 31-day-old piglets. Light microscopy of the intestinal morphology at different sections of the gastrointestinal tract. BF, breastfed; SF, standard formula; EF, experimental formula with milk fat globule membrane supplementation.

Morphological analyses were conducted on the duodenum, jejunum, ileum, and colon of the 31-day-old piglets (Figure 1). The piglets in the SF group exhibited significantly reduced villus height, villus base width, and villus surface area in the jejunum compared to those of piglets in the BF group (Table 4). No differences between the EF and BF groups were observed, nor were there differences in other parts of the small and large intestines among the groups.

**Table 4:** Effects of dietary type on the intestinal morphology of 31-day-old piglets (*n* = 4–6).

Item	Group		
	BF	SF	EF
Jejunum			
Villus height, μm	267 ± 56.16^a^	171.5 ± 56.96^b^	212.8 ± 29.08^a,b^
Villus base width, μm	61.64 ± 4.90^a^	39.78 ± 10.84^b^	56.83 ± 10.53^a,b^
Villus surface area, μm^2^	55,010 ± 26,630^a^	23,547 ± 11,169^b^	41,612 ± 12,555^a,b^

^a,b^*p* < 0.05. BF, breastfed; SF, standard formula; EF, experimental formula with milk fat globule membrane supplementation.

### 3.3 Microbial distribution across intestinal regions

**Figure 2:**
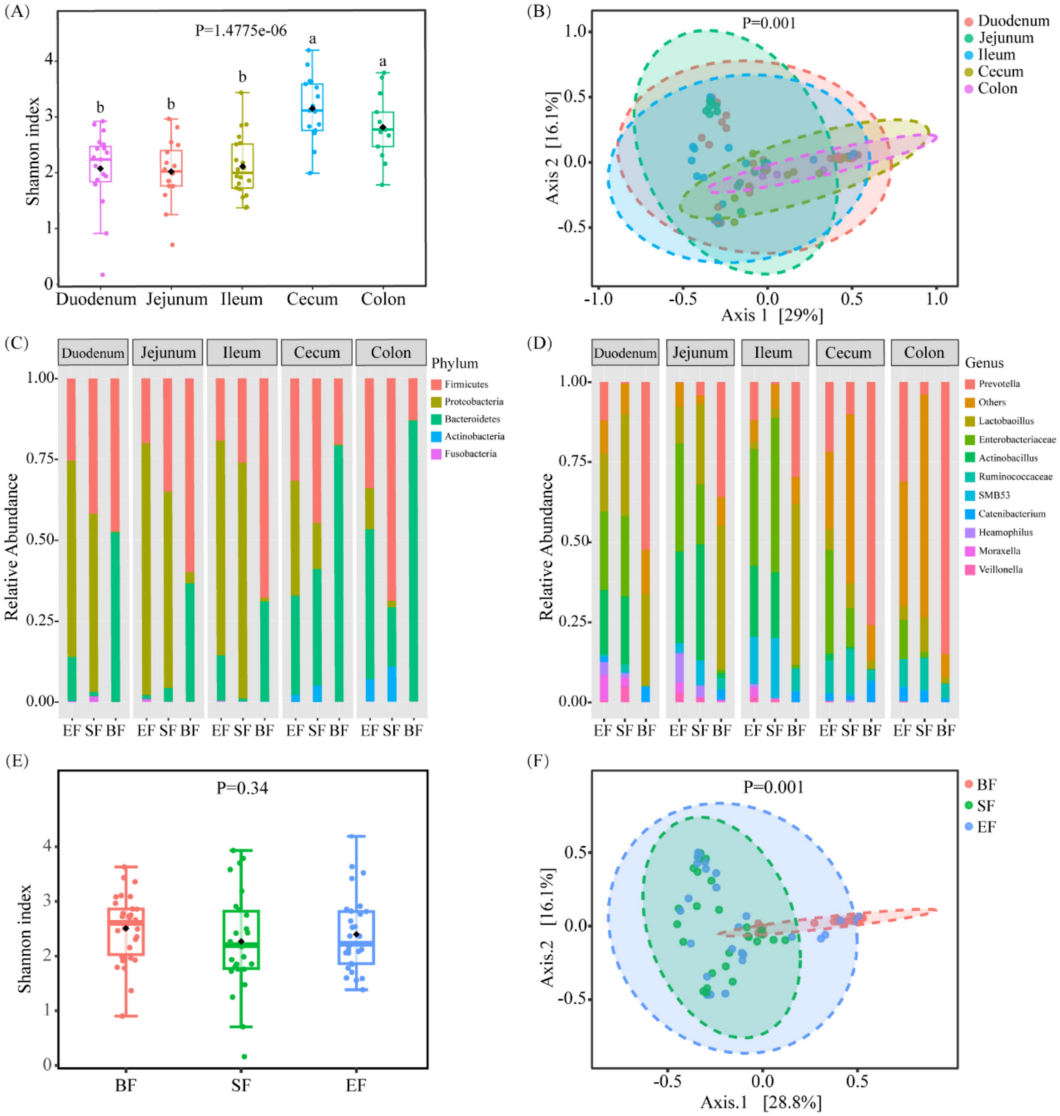
Bacterial community variation across discrete gut locations *n* = (13–20) and the effect of different dietary types on the intestinal microbiota of 31-day-old piglets (*n* = 26–30). **(A)** Alpha and **(B)** beta diversities of bacterial communities at discrete gut locations. **(C,D)** Microbial composition at the phylum **(C)** and genus **(D)** levels in the intestinal tracts of 31-day-old piglets. **(E)** Alpha and **(F)** beta diversities of gut microbiota in piglets fed different dietary regimens. BF, breastfed; SF, standard formula; EF, experimental formula with milk fat globule membrane supplementation. Data were presented as the means ± SD. ^a,b^ *p* < 0.05.

To assess the phylogenetic diversity of microbial communities across various sections of the gastrointestinal tract, 16S rRNA gene sequencing was employed. This analysis enabled a comprehensive comparison of both *α*- and *β*-diversity indices for microbial populations sampled from five distinct anatomical regions: the duodenum, jejunum, ileum, cecum, and colon. The large intestine, comprising the cecum and colon, exhibited significantly elevated Shannon diversity indices—an indicator that integrates both species richness and distribution uniformity—relative to the duodenum, jejunum, and ileum (*p* < 0.05; Figure 2A). No substantial variation in *α*-diversity was noted among the three small intestinal segments. With respect to *β*-diversity, principal coordinate analysis (PCoA) based on Bray–Curtis distance metrics revealed a clear segregation between microbial communities in the small and large intestines (Figure 2B), with most samples clustering distinctly according to their intestinal location.

Further taxonomic profiling was performed to delineate compositional differences in microbial populations across gut regions at both the phylum and genus levels. In the small intestine, dominant phyla included *Proteobacteria*, *Firmicutes*, and *Bacteroidetes* (Figures 2C,D). In contrast, samples from the cecum and colon demonstrated a decreased prevalence of *Proteobacteria* and an increased abundance of *Bacteroidetes*. At the genus level, key bacterial taxa in the small intestine included *Prevotella*, *Enterobacteriaceae*, *Lactobacillus*, *Actinobacillus*, and several unclassified genera. Notably, *Prevotella* was more prevalent in the cecum than in the small intestine, while *Enterobacteriaceae* were less abundant.

### 3.4 Influence of diet on gut microbial profiles

To elucidate the impact of dietary interventions on gut microbial composition, 16S rRNA sequencing data were stratified by feeding groups. No significant differences in Shannon diversity were observed among groups (Figure 2E). However, PCoA based on Bray–Curtis dissimilarities revealed distinct microbial community structures, particularly between the breastfed (BF) group and the two formula-fed groups (SF and EF) (Figure 2F, *p* < 0.05). In terms of predominant genera, the BF group was enriched in *Prevotella* and *Lactobacillus*; the SF group exhibited elevated levels of *Lactobacillus*, *Enterobacteriaceae*, and *Actinobacillus*; while the EF group shared a composition dominated by *Prevotella*, *Enterobacteriaceae*, and *Actinobacillus*. Further site-specific analysis revealed a significantly higher abundance of *Oxalobacter* and *Pasteurella* in the cecum of the SF group compared to the other groups (LefSe, *p* < 0.05; Supplementary Table 1).

### 3.5 Diet-induced modulation of serum metabolite profiles

**Figure 3:**
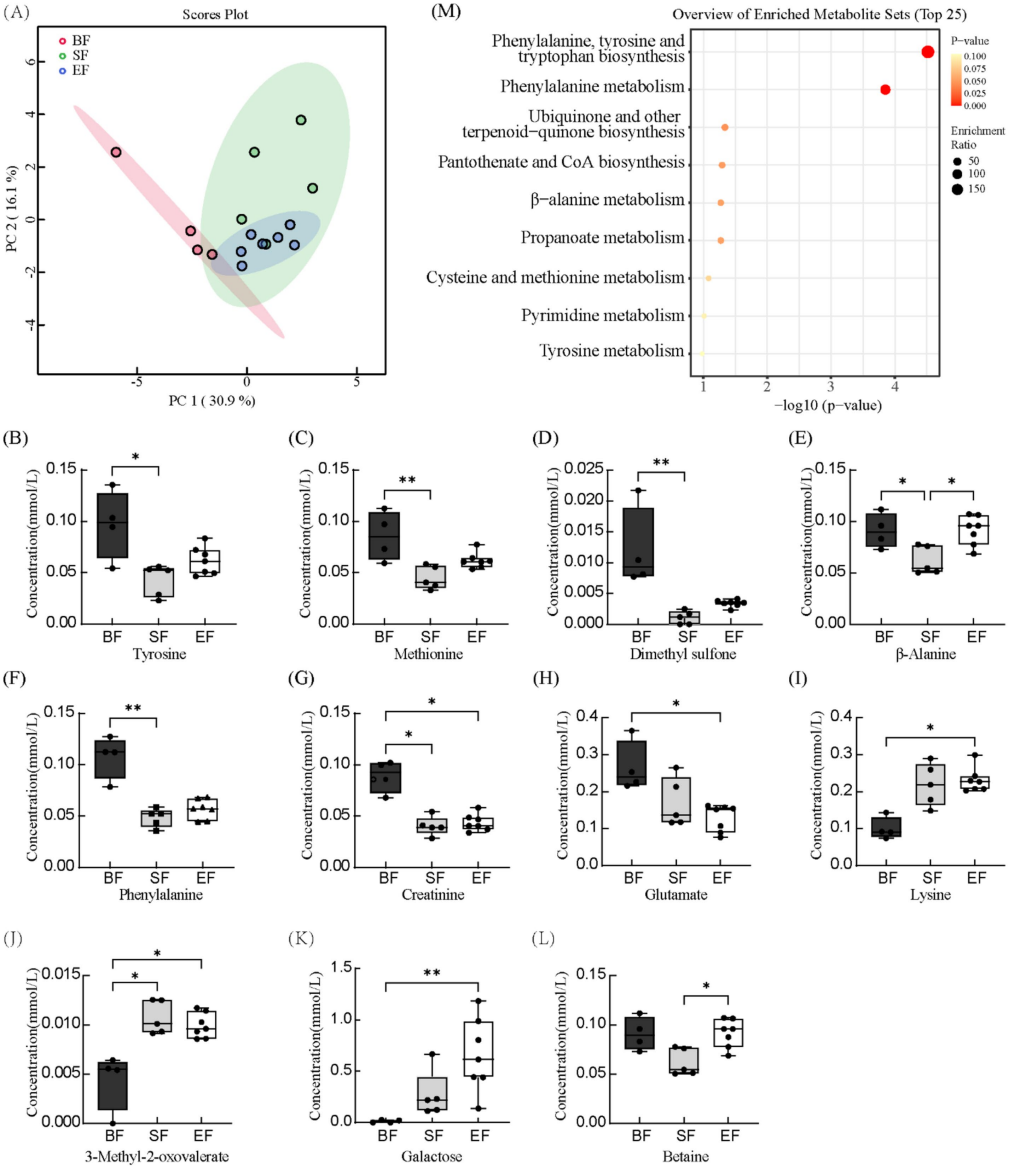
The effects of different diet types on serum metabolites in 31-day-old piglets (*n* = 4–7). **(A)** Partial least-squares discriminant analysis score plot of serum metabolome samples collected from 31-day-old piglets. **(B–L)** Effect of dietary type on serum metabolite concentrations. **(M)** Enriched metabolite sets in the BF group compared to the SF group. BF, breastfed; SF, standard formula; EF, experimental formula with milk fat globule membrane supplementation. Data were presented as the means ± SD (*n* = 5–7 per group). *p < 0.05 indicates a difference between the two groups; ** *p* < 0.01 indicates an extremely significant difference between the two groups.

Proton nuclear magnetic resonance (^1^H-NMR) spectroscopy was applied to characterize serum metabolite profiles in 21- and 31-day-old piglets based on dietary intervention. Across all groups, 67 distinct serum metabolites were identified. Samples collected prior to dietary differentiation displayed similar metabolic signatures and were therefore excluded from further comparative analyses. At day 21, metabolite concentrations were assessed using the Kruskal–Wallis test (Supplementary Figure 1). Levels of betaine, carnitine, and inositol were significantly elevated in the BF group relative to the SF group; however, no significant differences were detected between the BF and EF groups for these compounds. At day 31, principal component analysis revealed pronounced differences in the metabolic profiles of the BF group compared to the formula-fed groups (SF and EF), while no distinct separation was evident between SF and EF groups (Figure 3A). Further statistical interrogation using the Kruskal–Wallis test identified several metabolites with significant dietary associations. Concentrations of tyrosine, methionine, dimethyl sulfone, *β*-alanine, phenylalanine, and creatinine were significantly reduced in the SF group compared to the BF group (Figures 3B–G). In contrast, the level of 3-methyl-2-oxovalerate was elevated in the SF group (Figure 3J). Additionally, in the EF group, glutamate and creatinine were reduced, while lysine, 3-methyl-2-oxovalerate, and galactose were increased compared to BF piglets (Figures 3G–K). The EF group also exhibited higher *β*-alanine and betaine concentrations than the SF group (Figures 3E,L). Finally, enrichment pathway analysis indicated significant downregulation of phenylalanine, tyrosine, and tryptophan biosynthesis, as well as phenylalanine metabolism, in the SF group relative to BF (Figure 3M).

### 3.6 Analyses of differential metabolites and distinct microbial taxa

**Figure 4:**
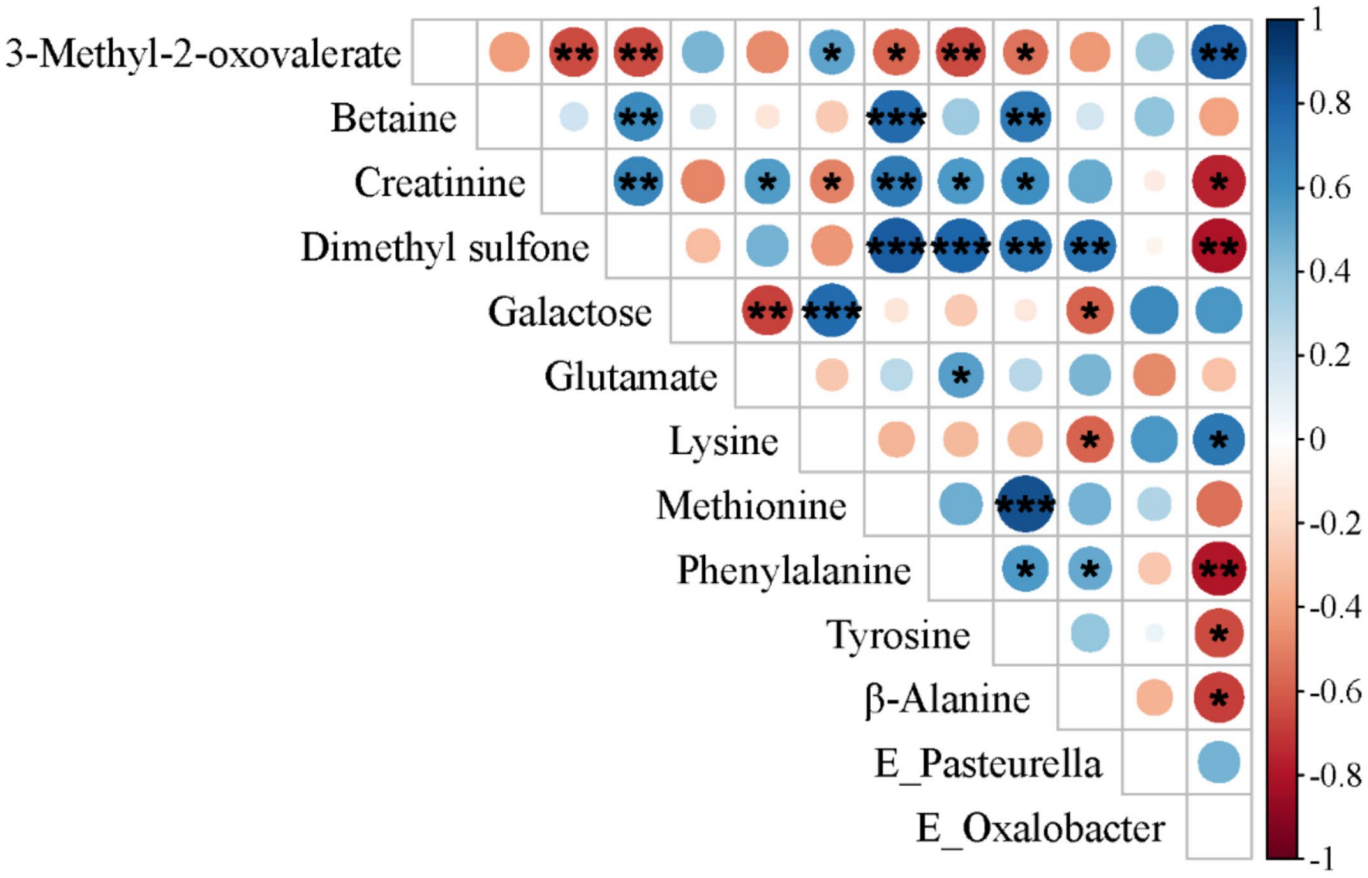
Spearman’s correlation analysis between differentially metabolized substances and distinct microbial communities in 31-day-old piglets (*n* = 17). *p < 0.05; **p < 0.01; ***p < 0.001.

To further elucidate the influence of the gut microbiota on metabolic status, we performed Spearman’ correlation analysis to assess associations between differentially abundant metabolites and differentially represented microbial taxa. In the SF group, a significant negative correlation was observed between *Oxalobacter*, which was significantly enriched in the cecum, and creatinine, dimethyl sulfone, phenylalanine, tyrosine, and β-alanine, but a significant positive correlation was observed with 3-methyl-2-oxovalerate and lysine (see Figure 4).

## 4 Discussion

Diet plays a pivotal role in establishing the infant gut microbiota (34), with feeding strategies exerting direct influence on microbial composition by supplying substrates that support bacterial growth and activity (35). Breastfeeding offers a wide array of advantages, including enhanced protection against infections (36, 37), a lower probability of developing obesity (38, 39), and a reduced incidence of allergic responses (40). However, breastfeeding is not always a feasible option for all caregivers (41). In such cases, infant formula provides an essential alternative. Conventional formulas often substitute dairy lipids with vegetable oils, which lack the milk fat globule membrane (MFGM), a bioactive structure inherent to human milk. MFGM has been shown to support multiple aspects of infant development, including physical growth, intestinal maturation (8), immune system function (42), and microbial colonization in the gut (43). In this investigation, we demonstrated that piglets consuming the MFGM-enriched experimental formula (EF) exhibited intestinal development, microbial profiles, and metabolic features comparable to those observed in breastfed (BF) piglets.

The small intestine serves as the principal site for digestion and absorption of nutrients in mammals (44). Enterocytes residing within the mucosal layer of the small intestine absorb and transport nutrients, while also participating in immune defense, barrier maintenance, fluid-electrolyte homeostasis, and the production of various proteins. Proper intestinal development and the proliferation and differentiation of epithelial cells are foundational for maintaining barrier integrity. The protective effects of MFGM on intestinal barrier function have been extensively studied (8, 45). For example, supplementation with MFGM in neonatal rats increased the numbers of goblet, enteroendocrine, and Paneth cells in the jejunum (27). In alignment with these findings, our study found that piglets in both BF and EF groups exhibited significantly greater villus height and base width in the jejunum compared to those in the standard formula (SF) group, resulting in an expanded villus surface area. Previous studies have demonstrated that dietary fructose elongates intestinal villi by prolonging enterocyte survival. The specific mechanism involves fructose-1-phosphate (F1P), a metabolite of dietary fructose, which inhibits the M2 isoform of pyruvate kinase (PKM2). This inhibition significantly enhances the survival rate of hypoxic intestinal cells, specifically those located at the villus tip (46). Furthermore, inadequate dietary protein intake and chronic stress have also been shown to induce significant villus elongation (47, 48). Since the conditions in the present study preclude both protein insufficiency and chronic stress, future research should prioritize investigating whether dietary MFGM supplementation enhances enterocyte survival by inhibiting PKM2, or potentially via an analogous mechanism. These outcomes reinforce earlier evidence suggesting that MFGM contributes to intestinal development, epithelial proliferation, and cellular differentiation. Furthermore, a previous study on MFGM-mediated enhancement of intestinal barrier function in piglets revealed that MFGM intervention during the first week after birth significantly upregulated the gene expression of ileal tight junction proteins (E-cadherin, occludin, claudin-4, and zonula occludens-1), mucins (mucin-13 and mucin-20), and interleukin-22 (IL-22) (8). This finding indicates that MFGM intervention strengthens both the physical and chemical barriers of the intestine, thereby protecting against pathogenic infections.

Despite the advantages of using fecal samples—primarily due to their non-invasive nature—gut microbiome research increasingly recognizes the need to assess microbial populations across various anatomical sites for a more comprehensive understanding of host–microbe interactions. Studies have shown that localized sampling across different intestinal regions can more effectively identify niche-specific microbial symbionts (49). Therefore, in the current study, we collected samples from both the small intestine (duodenum, jejunum, ileum) and the large intestine (cecum, colon) to characterize how MFGM supplementation differentially influences microbial community structures throughout distinct gastrointestinal environments.

The phospholipid components of MFGM enhance the adhesion of *Lactobacillus* to epithelial cells, such as Caco-2 and goblet cells, by altering cellular membranes, thereby increasing their retention time within the gastrointestinal tract (50). *In vitro* studies using Caco-2 cells have also shown that MFGM-derived gangliosides can inhibit the binding of enterotoxigenic and enteropathogenic *Escherichia coli* (51). Additionally, MFGM fosters colonization by health-promoting bacteria like *Ruminococcus*, known for producing short-chain fatty acids, and *Bifidobacterium* (8, 19, 52). The antimicrobial functions of MFGM operate through two primary mechanisms: (1) impeding pathogen adherence to epithelial surfaces via a decoy effect, and (2) exerting direct bactericidal actions. While we observed no significant differences in total bacterial counts between BF and formula-fed groups, notable differences in microbial community structures were detected, consistent with previous studies (53–56). Specifically, the cecum of SF piglets harbored a significantly elevated abundance of *Oxalobacter formigenes*, a bacterium that degrades oxalate and may reduce the risk of calcium oxalate kidney stone formation (57, 58). However, the SF group also exhibited an increased presence of *Pasteurella*, raising concern due to its pathogenic potential (59). Among *Pasteurella* species, *P. multocida* type D produces a potent toxin (PMT), which has been shown to induce severe organ damage and mortality in pig and mouse models (60). Importantly, dietary supplementation with synbiotics—including probiotics and xylo-oligosaccharides—has been reported to lower *Pasteurella* levels in piglets (61). In the present study, EF piglets displayed a significantly lower abundance of *Pasteurella* compared with their SF piglets, which is consistent with the well-documented antimicrobial properties of MFGM. The reduction of *Pasteurella* in the EF group may be attributed to several complementary mechanisms. First, the phospholipids and membrane-associated glycoproteins abundant in MFGM are capable of directly limiting pathogen adhesion and colonization (62). Second, MFGM and its bioactive components—including lactoferrin, sialic acid, and phospholipids—facilitate the enrichment of beneficial taxa such as Bifidobacterium, Lactobacillus, and short-chain fatty acid (SCFA)-producing bacteria (e.g., Ruminococcaceae, Roseburia, and Akkermansia) (19, 63, 64), which in turn may suppress *Pasteurella* through competitive exclusion. Third, MFGM contributes to the maturation of intestinal barrier functions by reinforcing epithelial tight junctions, enhancing mucin (MUC2)-based protective layers, and stimulating immune defenses such as antimicrobial peptides and secretory IgA (8). Collectively, these effects foster a more resilient intestinal microenvironment that restricts the colonization and expansion of pathogenic bacteria.

The infant gut microbiota is integral to nutrient metabolism, particularly through the synthesis of essential metabolites such as amino acid derivatives, vitamins, and short-chain fatty acids, which are not endogenously produced by the host. It also enhances the digestive capacity of the intestine (42). Previous metabolomic studies have shown marked differences in metabolic profiles between BF and SF infants, with the former favoring lipid metabolism and the latter more reliant on protein pathways (13). Carnitine, which facilitates mitochondrial fatty acid transport, is essential for lipid utilization. In our results, BF piglets exhibited higher serum concentrations of carnitine, betaine, and inositol at day 21 compared to SF piglets, consistent with the lipid-oriented metabolic phenotype observed in breastfed infants (13). Phosphatidylcholine in MFGM is the primary carrier of arachidonic acid and docosahexaenoic acid in plasma (65). EF piglets also displayed elevated betaine levels relative to SF piglets, and these concentrations were similar to those in BF piglets at both days 21 and 31, suggesting that MFGM can realign the metabolic phenotype of formula-fed piglets toward that of BF piglets. Betaine is a major metabolite of choline. The elevated betaine levels in EF piglets suggest that MFGM supplementation may help alleviate liver injury, restricted lean body mass gain, and impaired development of the lungs and nervous system caused by choline deficiency (65). Additionally, rapidly growing infants exhibit high demand for choline, which is primarily utilized to support phosphatidylcholine (PC) synthesis for substantial tissue growth and betaine-mediated one-carbon metabolism (66). A study in rats demonstrated that female offspring from prenatal choline-supplemented dams exhibited higher insulin sensitivity during the weaning period compared to the control group, which may facilitate energy provision for rapidly growing infant tissues (e.g., brain, muscle) (67). Betaine also promotes hepatic lipid metabolism by increasing mitochondrial content (68).

The elevated betaine in BF piglets is likely due to the high choline content of breast milk (69), and MFGM-supplemented foods have been reported to raise serum choline levels (12), likely through their phosphatidylcholine content. Therefore, based on the findings of this study, we speculate that the modulation of metabolic differences between breastfed and formula-fed infants by MFGM may be achieved through the regulation of choline metabolism. Further *in vitro* experiments are required to validate this hypothesis. Indeed, MFGM-enriched formulas have been shown to reduce disparities in lipid metabolism between BF and SF infants by 12 months of age (70). The mechanisms potentially involved in this effect, as indicated by our research findings, include: (1) Phosphatidylcholine from MFGM serving as the primary carrier for arachidonic acid and docosahexaenoic acid in plasma, thereby facilitating lipid metabolism; (2) Betaine enhancing hepatic lipid metabolism by increasing mitochondrial content; (3) Dietary choline supplementation improving infants’ insulin sensitivity to promote energy provision. Pathway enrichment analysis of metabolic data from 31-day-old piglets revealed that phenylalanine, tyrosine, and tryptophan biosynthetic pathways were significantly downregulated in the SF group. Since mammals lack endogenous enzymes to produce these aromatic amino acids (71), their synthesis depends on microbial enzymatic activity. The observed pathway suppression in SF piglets was associated with a reduction in microbial taxa encoding the necessary biosynthetic enzymes. Correlation analysis further revealed lower serum concentrations of creatinine, dimethyl sulfone, phenylalanine, tyrosine, and *β*-alanine in SF piglets compared to BF piglets, with these reductions showing negative associations with Oxalobacter abundance. Conversely, 3-methyl-2-oxovalerate, which was elevated in SF piglets, exhibited a positive correlation with Oxalobacter. These findings emphasize the influence of formula composition on gut microbiota-driven metabolic outputs.

This study has several limitations. First, 16S rRNA sequencing constrains taxonomic identification to the genus level, limiting resolution. Second, the use of targeted metabolomics restricted our analysis to a subset of known primary metabolites. Future investigations should employ metagenomic sequencing and non-targeted metabolomics to provide a more comprehensive view of how MFGM supplementation alters gut microbial ecosystems and metabolic networks in early development.

## 5 Conclusion

Despite physiological distinctions in perinatal intestinal development between neonatal piglets and human infants, the present study validates piglets as a viable model for assessing dietary interventions in pediatric nutrition. Supplementing the formula with MFGM enhanced the intestinal architecture, microbial community composition, and metabolic outcomes in piglets, closely mirroring phenotypes observed in their BF counterparts. Compared to BF piglets, those fed SF exhibited significant reductions in jejunal villus height, crypt depth, and mucosal surface area, coupled with cecal enrichment of *Oxalobacter* and *Pasteurella* genera. Notably, these aberrations were absent in piglets receiving MFGM-fortified formula (EF group). Furthermore, EF piglets demonstrated elevated serum betaine concentrations compared to SF animals. Overall, this study suggests that MFGM supplementation may mitigate metabolic disparities between breastfed and formula-fed neonates through dual modulation of choline metabolism and gut microbiota.

## Data Availability

The original contributions presented in the study are publicly available. This data can be found here: https://ngdc.cncb.ac.cn/gsa/search?searchTerm=CRA030441.
