# Supplementary material for: Milk fat globule membrane supplementation in formula modulates the gut microbiome and metabolic status of piglets and normalizes intestinal development
Source: Front Nutr. 2025 Oct 3;12:1632519. doi: 10.3389/fnut.2025.1632519 (PMC12531032; doi:10.3389/fnut.2025.1632519)
Supplement: Supplementary file 1 [file Table_1.DOCX]

**Supplementary Table 1** LefSe analysis of the microbial community in cecal contents

| name | P_values | FDR | BF | SF | EF | LDAscore |
| --- | --- | --- | --- | --- | --- | --- |
| Oxalobacter | 0.006889 | 0.043514 | 1720.9 | 49343 | 2474.6 | 4.38 |
| Pasteurella | 0.007252 | 0.043514 | 2.5815 | 31579 | 5963.7 | 4.2 |
| Prevotella | 0.012633 | 0.050531 | 381820 | 0 | 14199 | 5.28 |
| Synergistes | 0.017692 | 0.053077 | 558.14 | 8821.6 | 1325.5 | 3.62 |
| Dorea | 0.025965 | 0.062316 | 66315 | 0 | 0 | 4.52 |
| Cronobacter | 0.03811 | 0.07622 | 1118.9 | 10724 | 5246.2 | 3.68 |
| Anaerotruncus | 0.048346 | 0.082879 | 947.49 | 7929 | 593.3 | 3.56 |
| Bacteroides | 0.095255 | 0.12917 | 348870 | 712690 | 790760 | 5.34 |
| CF231 | 0.096876 | 0.12917 | 35000 | 20946 | 28959 | 3.85 |
| Not_Assigned | 0.22454 | 0.26945 | 60155 | 58330 | 63251 | 3.39 |
| Lactobacillus | 0.33265 | 0.34596 | 33402 | 2869 | 5586.8 | 4.18 |
| Eubacterium | 0.34596 | 0.34596 | 70091 | 96767 | 81646 | 4.13 |

BF, breastfed; SF, standard formula; EF, experimental formula with milk fat globule membrane supplementation.


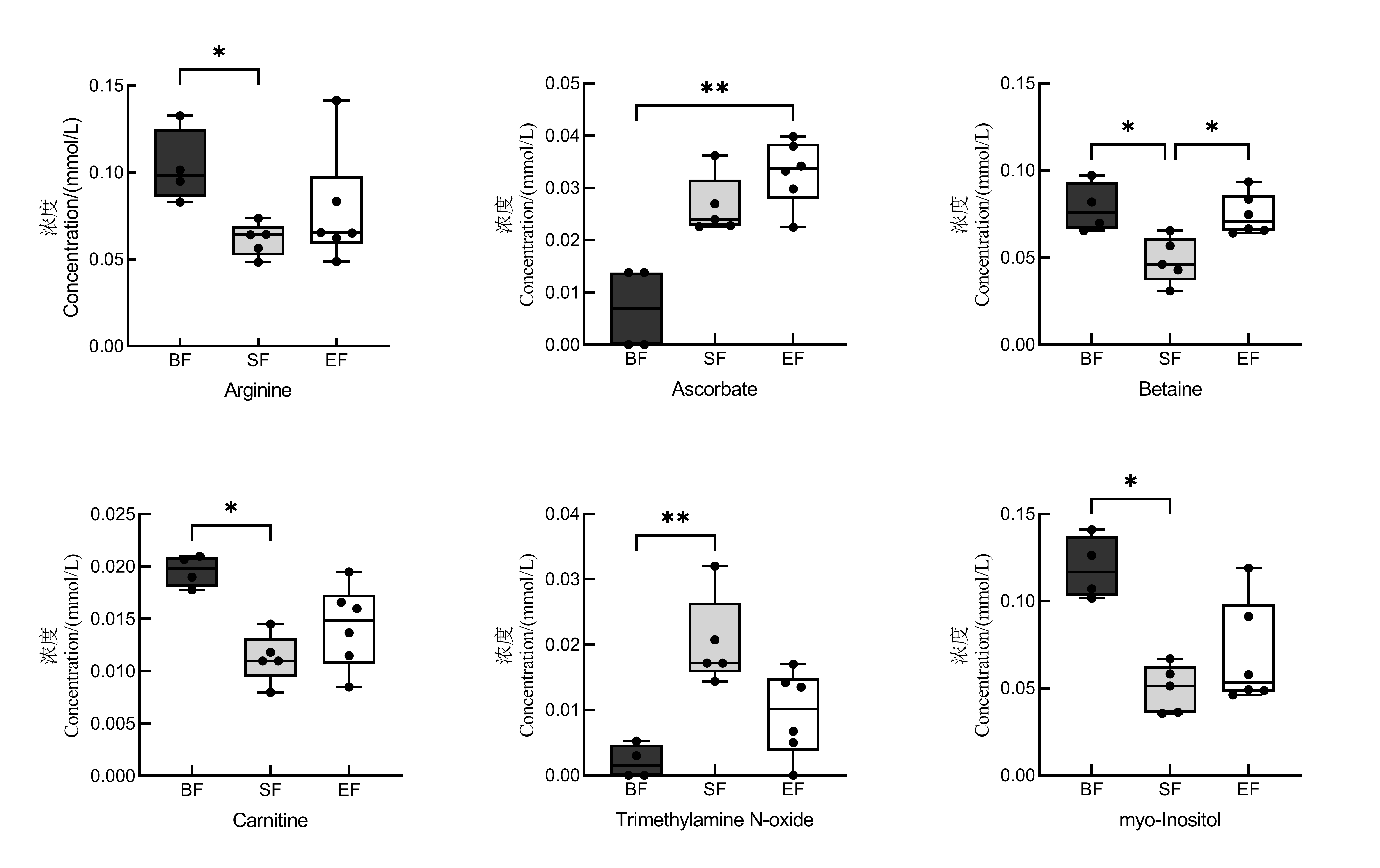


**Supplementary Figure 1:** Differential metabolites in 21-day-old piglets fed different dietary types. BF, breastfed; SF, standard formula; EF, experimental formula with milk fat globule membrane supplementation. * P <0.05; ** P <0.01
